# Giant Partially Thrombosed Coronary Aneurysm in Multisystem Inflammatory Syndrome Associated with SARS-CoV-2 in Children

**DOI:** 10.1155/2022/3785103

**Published:** 2022-09-28

**Authors:** Karen Daniela Manchola Narváez, Natalia del Pilar Delgado Ortíz, Iván José Ardila Gómez, Pilar Pérez López, Martín Fernando Rivera Ortíz

**Affiliations:** ^1^Pediatric Resident, Surcolombiana University, Hospital Universitario de Neiva, Neiva Huila, Colombia; ^2^Ivan Jose Ardila Gomez, Pediatric Critical Care, Clínica Uros, Hospital Universitario de Neiva, Surcolombiana University, Neiva-Huila, Colombia; ^3^Pilar Pérez López, Pediatric Rheumatologist, Clínica Uros, Clínica Medilaser, Hospital Universitario de Neiva, Surcolombiana University, Neiva-Huila, Colombia; ^4^Pediatric Cradiologist, Pediatric Congenital Cardiac Sonographer, Hospital Universitario de Neiva, Neiva, Surcolombiana University, Neiva, Colombia

## Abstract

Multisystem inflammatory syndrome in children (MIS-C) is a postinfectious condition which usually develops 4 to 6 weeks after SARS-CoV-2 infection in a genetically predisposed individual. Clinical features are heterogeneous and include fever, respiratory compromise, mucocutaneous involvement with conjunctival abnormalities and erythematous exanthem, abdominal pain, and diarrhea. Neurologic and cardiovascular symptoms can also develop, including coronary artery dilatation. Some cases involve 2 or more organs and require critical admission. Echocardiography is the mainstay of cardiac evaluation in the acute setting as well as on outpatient follow-up. We present the case of a 4-month-old female with no past medical or surgical history who presented with a prolonged febrile syndrome associated with severe respiratory illness, gastrointestinal symptoms, and mucocutaneous abnormalities. Diagnosis of MIS-C was established based on clinical findings, persistently elevated markers of systemic inflammation and positive SARS-CoV-2 molecular test and evidence of prior SARS-CoV-2 infection with SARS-CoV-2 IgG positive. Echocardiogram evidenced myopericarditis and coronary aneurysms and patient was deemed candidate for immunomodulatory therapy with intravenous immunoglobulin (IVIg), resulting in favorable clinical and paraclinical outcomes. Few cases of giant coronary aneurysms have been reported in children. There are no existing literature reports about coronary thrombosis or thrombus formation resulting from vascular aneurysmal dilations in this population. As such, the prognosis and natural history of coronary artery aneurysms in the setting of MIS-C remain largely unknown.

## 1. Introduction

COVID-19, initially known as a cause of severe acute respiratory syndrome, has represented a major challenge for those working in pediatric healthcare, where a great diversity of clinical manifestations has been documented. During the onset of the COVID-19 pandemic, the first observational studies showed that most children were asymptomatic [1] or were not severely affected [2].

However, in the United Kingdom in April 2020, a potentially fatal hyperinflammatory condition was first described. This condition shared clinical characteristics with incomplete Kawasaki disease, toxic shock syndrome, and macrophage activation syndrome, consistent of a febrile syndrome associated with mucocutaneous involvement, polymorphic rash, edema and erythema on palms and soles, coronary involvement, and elevation of inflammatory markers [3–5]. This condition called Multisystemic Inflammatory Syndrome in children (MIS-C) is recognized as a complication that generally develops 4 to 6 weeks after infection by SARS-CoV-2 [1], a situation suggesting that the virus may be a trigger for genetically predisposed individuals. 53–80% of them did not show positivity in the polymerase chain reaction test (RT-PCR), but the vast majority, from 75% to 90%, had serological positivity IgG or an epidemiological link with the infection [1, 6].

The overall proportion of children with SARS-CoV-2 infection presenting with MIS-C is largely unknown. Most patients do not have past medical conditions and the mean age of presentation is around 8 years [1]. Its clinical manifestations include fever, respiratory involvement in 30–70%, mucocutaneous involvement in 74%, conjunctival changes in 45–54%, erythematous rash in 52–62%, in addition, gastrointestinal manifestations such as abdominal pain, vomiting, and diarrhea in 53–92%, neurological and cardiovascular symptoms in 80%. 71–90% of patients presented involvement of more than 2 organs and half of the patients require admission to the intensive care unit during their hospital stay [6–8].

Cardiac complications are significant, with more than 80% of patients presenting cardiac involvement and around 10% to 40% of cases developing alterations in the coronary arteries. Among those most documented include coronary hyperechogenicity and with a lower incidence, true coronary aneurysm [5, 6] which if not timely treated, could lead to fatal outcomes. Coronary artery aneurysm is a rare clinical entity defined by an abnormal focal dilatation of a coronary artery exceeding the adjacent normal segment in diameter by at least 1.5-fold [9]. The size threshold for a small aneurysm is defined by *Z* score ≥2.5 to <5, medium aneurysm with a *Z* score ≥5 to <10, and a giant aneurysm with a *Z* score ≥10 or ≥8 mm in diameter [10].

Echocardiography is the cornerstone for evaluation of cardiac involvement in the acute phase and during follow-up. The most common findings include left ventricular dysfunction and abnormalities in coronary arteries including dilatation. Reports of giant aneurysmal alterations are not common and to date there is no information on the presence of thrombi in coronary dilatations developed in association with this condition.

## 2. Clinical Case

A 4-month-old female patient presented with fever. She had no significant clinical history, weighing 8 kg (ideal 6.9 kg), height 64 cm, cephalic circumference 41 cm, BMI 19.5 and nutritional classification: *P*/*T Z* score: 1.65 Overweight risk, *T*/*E Z* score: 0.05 appropriate, BMI *Z* score: 1.63 Overweight risk, PC *Z* score: -0.31 Normal. She was admitted with a prolonged febrile syndrome of 15 days, associated with severe respiratory symptoms, liquid stools, and nonpurulent bilateral conjunctival injection. Given persistence of fever, elevated inflammatory markers (CRP and ESR), and a positive molecular test for SARS-CoV-2, further laboratory analyses were significant for leukocytosis and thrombocytosis, elevated D-dimer, elevated pro-BNP, and evidence of previous infection as indicated by positive antibodies to SARS-CoV-2 (Table 1). As an epidemiologic link to SARS-CoV-2 infection, she had contact with her grandfather who died from COVID-19. During physical examination, she had sustained tachycardia as a sign of low output, no hypotension, and appropriate respiratory pattern without requiring ventilator or vasoactive support, without neurological involvement.

Clinical and laboratory findings met WHO criteria for MIS-C. Despite the timeframe of the febrile condition and considering the laboratory findings, immunomodulatory therapy with intravenous (2 g/kg) was indicated along with aspirin (ASA) in anti-inflammatory dosage (50 mg/kg) in addition to anticoagulation with low molecular weight heparin (1 mg/kg) according to the recommendations described in the literature to date. A first echocardiogram demonstrated appropriate left ventricular contractility, with myopericarditis and aneurysmal dilatations of the left and right coronary arteries (Table 2). Electrocardiogram showed ST segment elevation in three continuous leads, a pattern suggestive of myocarditis.

Two days after starting immunomodulation with IVIG, the fever resolved, so corticosteroid treatment was not deemed necessary. She presented an appropriate clinical response, was discharged from the PICU 10 days later, and was discharged from the hospital 11 days after admission with anticoagulant therapy that she was already receiving in the hospital (1 mg/kg) and ASA in antiplatelet dose; she still receives treatment to date.T

**Figure 1:**
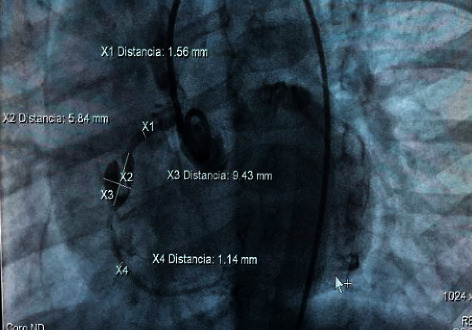
Cardiac catheterization: Aneurysm in the middle third of the right coronary artery, measuring 9.43 mm × 5.8 mm with an image of intracoronary thrombus occupying 2/3 of the aneurysmal lumen.

**Figure 2:**
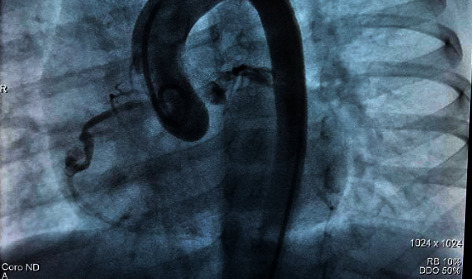
Cardiac catheterization: Aneurysm in the right coronary artery, with an image of intracoronary thrombus.

**Figure 3:**
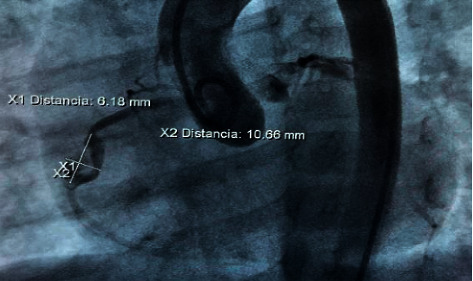
Cardiac catheterization: Intracoronary thrombus occupying 2/3 of the aneurysmal lumen in the middle third of the right coronary artery.

**Figure 4:**
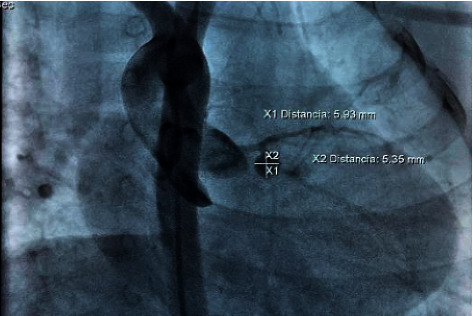
Cardiac catheterization: Aneurysm in the circumflex artery, measuring 5.9 mm × 5.3 mm without evidence of thrombus.

Echocardiograms performed during follow-up showed dilatation of the left cardiac chambers and persistence of aneurysmal dilatations. This led to a cardiac catheterization carried out 7 months after the onset of the inflammatory condition. A finding of an aneurysm in the middle third of the right coronary artery was described, measuring 9.43 mm × 5.8 mm with an image of intracoronary thrombus occupying 2/3 of the aneurysmal lumen. There was also evidence of an aneurysm in the circumflex artery, measuring 5.9 mm × 5.3 mm without evidence of thrombus (Figures 1–4).

## 3. Disscusion

MIS-C is a severe clinical condition with significant cardiac implications that occurs as a consequence of a dysregulated inflammatory response. It typically develops several weeks after acute SARS-CoV-2 infection, with a reported mortality of around 2% in high-income countries [4, 5]. In contrast, data from developing countries such as Colombia report mortality rates of 9% [11]. The incidence of cardiac biomarker elevation such as pro-BNP and troponin is 86.65% and 76.34%, respectively [12], on some occasions with extremely high levels as was with our current case, correlating with severe myocardial damage comparable to that from Kawasaki disease (KD). Significantly elevated levels of ferritin, procalcitonin, and D-dimer have also been documented, as in the case described [8].

Echocardiography has great diagnostic value when evaluating cardiovascular involvement. The main documented findings have been coronary artery dilatation by 17.83%, aneurysm by 6.85%, pericarditis by 20.97%, myocarditis by 35.97%, and myocardial dysfunction up to 68% of patients [6, 12]. Most of the described complications related to dilatation and coronary aneurysm are small with a *Z* score of 2–2.5, contrary to the case presented in which giant aneurysmal dilatations were found. This could probably be explained by young age, as with growth comes improved mechanical resistance of the internal elastic lamina to aneurysm formation [1].

Although infrequent, 3 cases described in Atlanta (Georgia) reported giant aneurysms occurring in children [13], however, so far there are no reports of aneurysmal thrombus formation. Despite the important presentation of MIS-C cases described, there are no national or Latin American publications about the findings of giant aneurysms, nor their evolution or complications.

Echocardiography plays an important role during longitudinal surveillance after MIS-C. Studies suggest that in patients with abnormal findings such as dilatations or aneurysms, it is necessary to carry out rigorous imaging monitoring, which in this case was not possible due to the difficulties with access to healthcare. The last outpatient echocardiogram report (8 months after the presentation of this hyperinflammatory syndrome) showed persistence of giant aneurysms in the right and circumflex coronary arteries [8].

The main purpose of treatment in MIS-C is to reduce systemic inflammation to protect affected organs and reduce or prevent complications such as coronary aneurysms and death. This treatment is guided according to the clinical presentation and requires a multidisciplinary team (cardiology, infectious diseases, rheumatology, hematology, and intensive care). To date, there are no randomized controlled clinical trials or comparative efficacy studies evaluating treatment strategies. This is why available guidelines are based on expert opinion, scientific societies, and previous experience with Kawasaki disease and other multisystemic disorders in children [2]. Immunomodulatory management with immunoglobulins continues to be the first choice, and has been prescribed as monotherapy or in conjunction with corticosteroids [7]. Other important interventions include administration of ASA in 58–74% of cases and anticoagulant management in 37–44%, based on the presence of serum markers [6].

The prognosis and natural history of coronary aneurysms associated with MIS-C are still uncertain. To date, reports show that even the subset patients with MIS-C requiring critical admission and/or those with severe cardiovascular involvement, recovery was seen without sequelae in 70–95% of cases. The prognostic impact on outcomes of those cases with aneurysmal thrombosis is not known, as this severe clinical presentation remains largely undescribed. This clinical entity requires further study and structured long-term follow-up due to the risk of progression of cardiac manifestations [2]. It has been established that in Kawasaki disease the risk of presenting thrombotic or stenotic complications is related to the size of the aneurysms; giant aneurysms (Z score ≥10) are the least likely to undergo resolution, and are associated with up to 50% risk of adverse outcomes such as thrombotic coronary occlusion, progressive stenosis requiring revascularization, or acute coronary syndrome within 30 years after the initial illness [10]. This highlights the importance of guaranteeing rigorous follow-up in order to anticipate early complications and carry out timely interventions.

## Figures and Tables

**Table 1:** Lab tests.

Lab Tests			
	21/09/2020	29/09/2020	MAY/2021
Complete blood count test (CBC)	Leucocytes: 22980 cel/uL (4–10 ^ 3/uL) Lymphocytes: 6320 cel/uL (0.8–4.0 10 ^ 3/uL) Neutrophils: 15030 cel/uL (2–710 ^ 3/uL) Hemoglobin: 8.1 g/dL (11-16) platelets: 1792000 cel/uL (150–450 10 ^ 3/uL)	Leucocytes: 11350 cel/uL (4–10 ^ 3/uL) Lymphocytes: 8240 cel/uL (0.8–4.0 10 ^ 3/uL) Neutrophils: 1850 cel/uL (2–7 10 ^ 3/uL) Hemoglobin: 8.6 g/dL (11–16) Platelets: 1224000 cel/uL (150–450 10^3/uL)	Leucocytes: 11230cel/uL (4–10 ^ 3/uL) Lymphocytes: 7740 cel/uL (0.8–4.0 10 ^ 3/uL) Neutrophils: 2600 cel/uL (2–7 10 ^ 3/uL) Hemoglobin: 11.8 g/dL (11–16) Platelets: 557000 cel/uL (150–450 10 ^ 3/uL)

CRP (C-Reactive Protein test)	13.59 mg/dL (<1)	0.6 mg/dL (<1)	

ESR (Erythrocyte sedimentation rate)	140 mm/h (0–10)	50 mm/h (0–10)	15 mm/h (0–10)

Troponin I	1.69 /mL (<0, 16)	7.97 ng/mL (<0, 16)	<0.16 ng/mL (<0, 16)

Pro-BNP	5691 /mL (<300)		372 pg/mL (<300)

D-dimer	4153 ng/mL (<500)	1625 /m L (<500)	

Ferritin	193.5 /mL (13–150)	151.7 /mL (13–150)	

Fibrinogen	615 mgs%	420 mgs%	

Clotting time	PT: 10.5 seg (9.5–12.9), PTT: 29.4 seg (21.6–29.2), INR: 0.96 (0.9–1.3).	PT: 11 seg (9.5–12.9), PPT: 24.4 seg, INR: 1.01 (0.9–1.3)	PT: 11.4 seg (9.5–12.9), PPT: 36.3 seg (21.6–29.2), INR: 1.06 (0.9–1.3).

RT-PCR SARS-CoV-2	Positive		

SARS-CoV-2 IgM	Positive		Negative

SARS-CoV-2 IgG	Positive		Positive

**Table 2:** Echocardiograms.

	Echocardiograms/Findings in coronary arteries
Day one from the diagnosis	^*∗*^Left coronary artery: Main: 0.32 cm (Z: 4.6). Medial descending: 0.53 cm (Z: 12). ^*∗*^Circumflex: 0.30 cm (Z: 5.5). ^*∗*^Right coronary artery: Proximal: 0.74 cm (Z: 17). Appropriate left ventricular contractile function and grade II to III pericardial effusion.

One month after diagnosis	^*∗*^Left coronary artery: Main: 0.51 cm (Z: 10). Proximal descending: 0.50 cm (Z: 11). Medial descending: 0.45 cm (Z: 10). Distal: 0.65 cm (Z: 16). ^*∗*^Proximal circumflex 0.44 cm (Z: 4.4). Distal: 0.62 cm (Z: 15). ^*∗*^Right coronary artery: Proximal coronary artery: 0.72 cm (Z: 16). Medial: 0.59 cm (Z: 14). Distal: 0.47 cm (Z: 11). Appropriate left ventricular contractile function and grade II pericardial effusion

Eight months after diagnosis	^*∗*^Left coronary artery: Main coronary artery: 0.35 cm (Z: 4.6). Proximal descending: 0.35 cm (Z: 6.3). Medial descending: 0.33 cm (Z: 7.4). Medial aneurysmal image: 0.95 × 0.91 cm (Z: 22.7). ^*∗*^Giant aneurysms in right medial and circumflex arteries. Preserved left ventricular contractile function.
